# Effect of green banana and pineapple fibre powder consumption on host gut microbiome

**DOI:** 10.3389/fnut.2024.1437645

**Published:** 2024-08-23

**Authors:** Chun Wie Chong, Mei Shan Liew, Weitze Ooi, Hassan Jamil, Angie Lim, Suet Li Hooi, Clarisse S. C. Tay, Gwendoline Tan

**Affiliations:** ^1^School of Pharmacy, Monash University Malaysia, Subang Jaya, Malaysia; ^2^Dole Specialty Ingredients, Dole Asia Holdings Pte., Ltd., Singapore, Singapore; ^3^AMILI, Singapore, Singapore

**Keywords:** green banana, pineapple, short chain fatty acids, gut microbiome, dietary fiber

## Abstract

**Purpose:**

To determine whether green banana powder (GBP) and pineapple fibre powder (PFP) promote beneficial bacterial species, directly improve human gut health and modulate the gut microbiome and understand their utility as functional foods and dietary supplements.

**Methods:**

Over 14 days, 60 adults followed protocol requirements, completed food diaries and study questionnaires, avoided consuming supplements with prebiotics, probiotics or postbiotics, and ingested food containing 5 g of total daily fibre [placebo (10.75 g), GBP (10.75 g) or PFP (7.41 g)]. Participants’ medical and baseline wellness histories, as well as stool samples, were collected at baseline, day 7 and 14. Stool DNA was processed for sequencing.

**Results:**

Dietary fibre and resistant starches (RS) in GBP and PFP promoted temporal increases in beneficial bacteria. GBP significantly elevated 7 species (*F. prausnitzii*, *B. longum*, *B. bifidum*, *B. adolescentis*, *B. pseudocatenulatum*, *B. obeum*, and *R. inulinivorans*), while PFP enriched 6 species (*B. ovatus*, *B. cellulosilyticus*, *B. bifidum*, *B. intestinalis*, *R. inulinivorans*, and *E. siraeum*). These bacteria, found to be deficient in younger adults, were promoted by both powders. PFP benefitted both genders aged 16–23, while GBP benefitted overweight/obese individuals, including females. GBP and PFP fiber and RS improved bowel regularity and health as well as metabolism by promoting histidine, branched-chain amino acids, short-chain fatty acids, and biotin production. The additional fiber caused “low” bloatedness and reduced “fairly bad” sleep disruptions, without affecting sleep durations.

**Conclusion:**

GBP and PFP supplementation increased beneficial bacteria and metabolites, improved host gut health, and present a valuable nutritional strategy for enhancing human health.

**Clinical trial registration:**

AMILI Institutional Review Board, Identifier 2023/0301.

## Introduction

The human gastrointestinal microbiome performs critical metabolic, immunologic, and protective roles (1, 2), and varies in composition along the digestive tract (3). Among the most abundant human gut phyla are *Bacteroidetes* and *Firmicutes*, while the most frequently detected genera include *Bacteroides*, *Bifidobacterium*, *Eubacterium*, and *Faecalibacterium* species (4). Gut microbiome composition and function are modulated by factors such as environment and dietary (5) fibre intake. Problems like constipation and reduced bowel regularity reflect the health and function of the gut (6) and its microbiome and are linked to increased risks of diseases such as chronic kidney disease and Parkinson’s disease. Dietary fibre improves bowel and gastrointestinal function by facilitating the movement of digested food through the gastrointestinal tract and increasing stool volume. It also contains fibre-bound bioactive compounds (e.g., antioxidants) that help to reduce glycaemia, oxidative stress, inflammation, and the risks of chronic diseases. In fact, dietary fibre is better at preventing cardiovascular disease than pharmaceutical interventions, and public health polices for disease prevention now recommend plant-rich diets (7).

Dietary fibres include digestible polysaccharides which serve as substrates for gut microbes and thus influence a host’s microbiome and immunity. Dietary fibres also include inulins and resistant starches (RS) that are bacteria-fermentable and soluble, and indigestible polysaccharides like fructooligosaccharides (FOS). Dietary fibre (8) and RS withstand digestion in the small intestine and are fermented by colon bacteria (9). Fermentable fibres can increase the diversity of microbes depleted by low-fibre Westernised diets (1) and may thus help to reduce the incidences of chronic diseases (e.g., type 2 diabetes). RS supports gastrointestinal health, benefits bowel cancers and cardiovascular diseases, reduces overall food intake, and help prevent, treat, or reduce the risk of chronic diseases (10, 11). Indigestible fibres confer prebiotic properties and health benefits through their fermentation end-products; in particular, short-chain fatty acids (SCFAs) like butyrate, propionate, and acetate. These SCFAs modulate antibody production, immune cell homeostasis and differentiation, appetite regulation and lipid metabolism (12). Butyrate, which colonocytes need for energy, are reduced by slow colon transits but increased by diets high in dietary fibres and RS which accelerate colon transit times. Butyrate also increase bioavailability of metals, protects the host from pathogens, and reduces chronic gut inflammation while increasing proteins that enhance gut barrier integrity and mucous production. Propionate is used in intestinal or hepatic gluconeogenesis for glucose homeostasis, while acetate is the most abundant SCFA in the circulation, protects from enteropathogenic infections, and can cross the blood-brain barrier.

Dietary supplementation with insoluble, non-fermentable fibres, such as that from bananas and pineapples, improves microbiome compositions and ameliorates disease. These popular, fibre-rich fruits are commonly grown as agricultural commodities in Southeast Asia and enriched in key bioactive compounds, nutrients, dietary fibre and RS (8, 9, 13). Their processing generates peel, pomace and stem waste or by-products. For example, banana processing leaves behind nearly 60% of its biomass as waste (14), which still retains nutrients including minerals, prebiotics, and dietary fibres (15). Recovery of fruit wastes and by-products reduces waste accumulation, pollution, and environmental problems (15, 16), while their reuse as health supplements and food ingredients may confer economic benefits (15).

Green bananas are often collected as an agricultural surplus (17), as their ripening is affected by environmental factors like temperature, humidity, and air circulation (18). Green bananas are rich in pectin, which slows gastric emptying, improves bowel function, reduces glucose and cholesterol absorption, and produces SCFAs when fermented by colonic microbiota. In turn, these processes reduce intestinal inflammation, improve gut microbiome balance, and lower the risk of inflammatory bowel disease (IBD). Green banana also contains RS (19, 20) which withstands digestion in the small intestine and may help to regulate insulin and blood glucose, reduce the size of subsequent meals, and serve as a prebiotic to modulate the microbiome (21). Animal studies of obesity showed that green bananas and their flours (GBF) regulated body weights and gut microbiota, and improved fat pad distribution, obesity-associated systemic inflammation (19), and metabolic statuses. GBF also recovered levels of *Clostridium*, *Bacteroides*, and *Parabacteroides* depleted by antibiotics (22). Additionally, GBF increased levels of bacteria that produce SCFAs, accelerating the recovery of gut microbiota and the repair of gut barriers.

Pineapple fibre is a prebiotic that stimulates the growth of beneficial microbiota including *Lactobacilli* and *Bifidobacteria* while also modulating their metabolism (23), promoting SCFA production, and improving lipid and cholesterol metabolism (16, 24–28). Pineapple waste and by-product powders contain non-digestible, insoluble fibres that gut bacteria ferment into antioxidant compounds. The powder promotes the growth of beneficial microbiota, including *Lactobacillus*, *Bifidobacterium*, *Firmicutes* and *Bacteroidetes*, while its faecal fermentation promotes propionate and acetate production (23). Pineapple powders also support probiotic organisms, especially *L. acidophilus*, *L. casei*, and *L. paracasei*, and enhance the antioxidant and antimutagenic properties of yogurt when added to it (16).

Green banana and pineapple consumption, therefore, provide multiple health benefits due to their impacts on the microbiome, and functional foods formulated with green banana powders (GBP) and pineapple fibre powders (PFP) may improve microbiome diversity. The World Health Organization has recommended a daily dietary intake of 25 g of fibre in adults, but exactly which fibre sources to consume is not well defined (20). As several bacterial species are associated with the beneficial effects of green banana (13, 19, 20, 22, 29) and pineapple (16, 23, 30), we thus conducted an interventional study to trace the abundance of beneficial bacterial species previously associated with GBP and PFP (Table 1). We also sought to determine if these species directly improve human gut health and modulate gut microbiome abundance and diversity, to understand their utility as functional foods and dietary supplements.

**Table 1:** Beneficial bacteria associated with banana and pineapple consumption and their putative functions.

No.	Bacteria species	Benefits	References
1	*Bacteroides ovatus*	Bacteria that can produce enzyme (lyase) to degrade cell wall	(31)
2	*Bacteroides cellulosilyticus*	Bacteria that can degrade glycans (complex carbohydrates)	(32)
3	*Akermansia muciniphila*	Bacteria that break down mucin (protein-carbohydrate complex) and produces acetate (SCFA)	(33)
4	*Faecalibaterium praunitzii*	Bacteria that produces butyrate (SCFA) anti-inflammatory metabolites	(34)
5	*Bifidobacterium longum*	Bacteria that can restore intestinal mucus layer (protective layer in intestine) and SCFAs	(35)
6	*Bifidobacterium bifidum*	Bacteria has produces bacteriocins (antimicrobial compound)	(36)
7	*Bifidobacteroum adolescentis*	Bacteria that can produce gamma aminobutyric acid (GABA) a neurotransmitter that helps neurological and mental health conditions	(23)
8	*Bifodobacterium pseudocatenulatum*	Bacteria that can reduce intestinal inflammation	(37)
9	*Bifodobacterium catenulatum*	Protects intestinal mucosal layer and prevent opportunistic bacteria enrichment	(38)
10	*Bacteroides intestinalis*	Bacteria that can degrade arabinoxylans (complex carbohydrates)	(39)
11	*Blautia obeum*	Bacteria that can degrade polysaccharide (complex carbohydrate)	(40)
12	*Roseburia inulinivorans*	Bacteria that utilizes inulin (soluble fibre) can produce butyrate and propionate (SCFAs)	(41)
13	*Alistipes onderdonkii*	Bacteria that has anti-inflammatory effect (transplant recipient)	(42)
14	*Eubacterium siraeum*	Bacteria that can degrade xylan (complex carbohydrate)	(43)
15	*Eubacterium rectale*	Bacteria that can break down dietary fibre	(44)

## Methods

### Population

**Figure 1:**
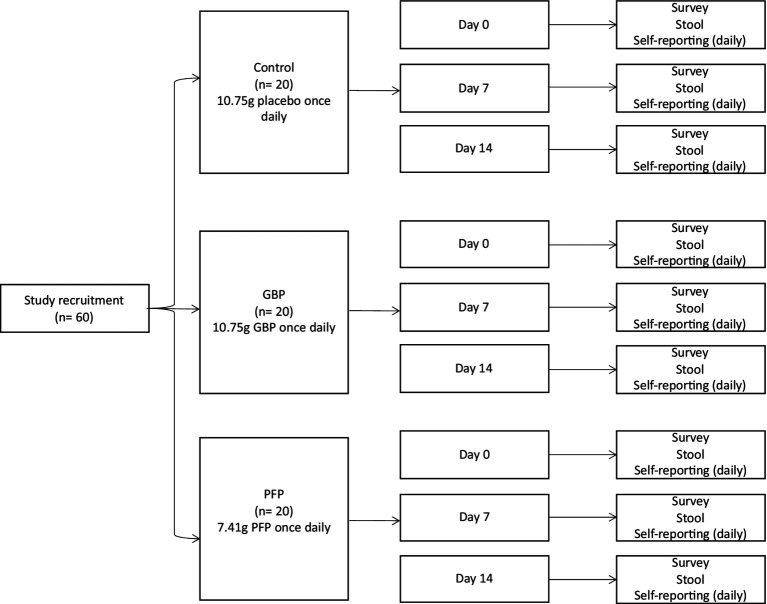
Study protocol of proposed human intervention trial.

We targeted a diverse population for nutritional supplementation analyses. Inclusion criteria were adults, male or female, between 21 to 65 years of age; body mass index (BMI) 18–29; ability and willingness to follow protocol requirements, complete food diaries and study questionnaires, avoid consumption of supplements with prebiotics, probiotics or postbiotics, and to swallow (deglutition). Participants were excluded by the principal investigator for use of oral antibiotics, antifungal and/or antiviral during the past 3 months; any clinically significant medical history, medical findings and/or ongoing medical or psychiatric conditions including malignancy, chronic metabolic diseases (i.e., diabetes, obesity, and liver disease) and gastrointestinal diseases (i.e., gastric ulceration and inflammatory bowel syndrome); use of long-term medicines or immunosuppressant drugs; known allergies to any of the study’s foods or fruits; smoking or consumption of tobacco products; pregnancy and lactation status. Informed consents were obtained via teleconferencing or in-person. For statistical power, 60 participants were included (Figure 1). The sample size was chosen based on the team’s previous study, which yielded statistically significant results (45). This study was approved by the AMILI Institutional Review Board, which adheres to the Declaration of Helsinki (AMILI IRB Ref: 2023/0301).

### Intervention

The intervention samples, GBP and PFP, were manufactured using advanced processing technologies that utilize surplus, waste, or by-products sourced from sustainable fruit farms (Dole Specialty Ingredients, Dole Asia Holdings Pte., Ltd., data on file). PFP production commences with the extraction of pineapple juice, resulting in a nutrient-rich pineapple pulp pomace. This pomace undergoes controlled drying to achieve optimal moisture content, followed by milling to attain particle sizes below 80 mesh, resulting in PFP. Similarly, unripe green bananas are processed to produce GBP through a systematic procedure involving sorting, washing, cutting, and drying the pieces to meet specific moisture content criteria. Rigorous control parameters are implemented to mitigate RS degradation, and the pieces are subsequently milled to achieve the requisite size characteristics, also below 80 mesh.

Participants (*n* = 60) were divided first into Japanese or non-Japanese ethnic groups, and then into control, GBP or PFP intervention groups. Over 14 days, participants consumed 10.75 g of placebo, 10.75 g of GBP or 7.41 g of PFP daily (both Dole Speciality Ingredients). The amount of fibre consumed was nutritionally designed to facilitate gut microbiome interactions. GBP or PFP were delivered as powders dissolved in any liquid of choice, to a total daily fibre intake of 5 g (Supplementary Table S1). Participants were required to avoid consuming other prebiotic or probiotic products but had no other dietary restrictions. Evaluations were conducted at day 0 (baseline), 7 and 14.

### Evaluations

At recruitment, participants’ basic demographic details, medical histories, and baseline wellness were recorded. Wellness was evaluated using the Pittsburgh Sleep Quality Index (PSQI), Perceived Stress Scale (PSS), Kessler Psychological Distress Scale (K10), and Bristol Stool Scale. Baseline dietary habits were recorded using the Food Frequency Questionnaire, and stool samples were collected using Zymo DNA/RNA shield (Zymo Research, Irvine, CA, United States) tubes, at baseline, day 7 and day 14. Post-intervention wellness was assessed using the PSQI, PSS, K10 and Bristol Stool Scales. Further validation was provided by the inclusion of participants’ daily reporting of food and fluid intake throughout the study period (day 0–14).

### Sample collection and analysis

#### Online questionnaires

Three questionnaires were administered at different time points via an online survey (Qualtrics, Provo, UT, United States). At baseline (day 0), Questionnaire A was used to collect data on the participants’ demographics, medical histories, and Gut Health Test, K10, PSS, PSQI, Stool Bristol Score and Frequency Questionnaire (FFQ) scores. On day 7, Questionnaire B (PSQI, Stool Bristol Score and bowel movement frequency) was used to collect participants’ data. On the last day of the study (day 14), Questionnaire C (K10, PSS, PSQI, Stool Bristol Score) was used.

#### Stool sample collection and processing

Stool (1 g) was collected and stored at −80°C until analysis. DNA was extracted using QIAmp PowerFecal Pro DNA extraction kits (Qiagen, Foster City, California, United States), and processed for shotgun metagenomic sequencing, with a minimum output and size per sample of 6GB at 40 million total reads.

### Data analysis

Data from questionnaire responses and metagenomic sequencing were de-identified. Sequencing data was processed using Biobakery3 workflow (46). Briefly, the microbial abundance and taxonomy were resolved using metaphlan3 and the functional pathway was annotated using humann3. The data was exported to R 4.2.2 and Python 3.7.10 for further analysis. Descriptive statistics were performed by comparing PSS, K10, PSQI, and Bristol Stool Scale, and nutrient scores across groups. Changes in alpha diversity between groups across time were compared using boxplot and the statistical significance were compared using Wilcoxon test (Supplementary Figure S1), with adjustments for differences in timepoints, gender, ethnicity, BMI, age and intervention group.

The abundance of selected beneficial bacterial species (Table 1) was compared based on (1) the proportion of subjects showing elevated abundance between T0 and T2, (2) cumulative log2 changes >1, and (3) negative binomial model after Cumulative Sum Scaling (CSS) normalisation using MaAsLin2 (47). To increase the robustness of the data, we only consider features significantly elevated in day 7 and day 14 (in comparison to day 0) and FDR <0.05 as significant for the MaAsLin2 analysis.

From the significant features, seven were selected for further analysis. We compared the functional pathways conferred by these bacteria. However, as the presence of genes does not represent the expression, the comparison was conducted to represent “functional potentials,” and a statistical test was not conducted. Additionally, the prevalence of these bacteria in the AMILI database (Supplementary Table S2, *n* = 669) was compared to identify demographics most likely to benefit from the supplementation of GFP and PFP.

## Results

### Participant demographics

The 60 participants recruited to non-Japanese or Japanese cohorts (Table 2) showed no differences in overall, average nutrient intakes between cohorts or experimental groups (GBP, PFP, control). Relative to the recommended fibre intake of 25 g/day, all participants demonstrated a low initial fibre intake.

**Table 2:** Demographic of participants enrolled in the pineapple and green banana study.

	Control (10 Japanese, 10 non-Japanese)	Pineapple (10 Japanese, 10 non-Japanese)	Green Banana (10 Japanese, 10 non-Japanese)
Mean age	40	38.5	42.2
Mean BMI	22.085	22.68	22.6
*Race*			
Japanese	10	10	10
Non-Japanese (Chinese)	10	9	9
Non-Japanese (Indian)	0	1	1
*Gender*			
Female	14	11	12
Male	6	9	8

### GBP and PFP improved bowel movement frequency and bowel health

**Figure 2:**
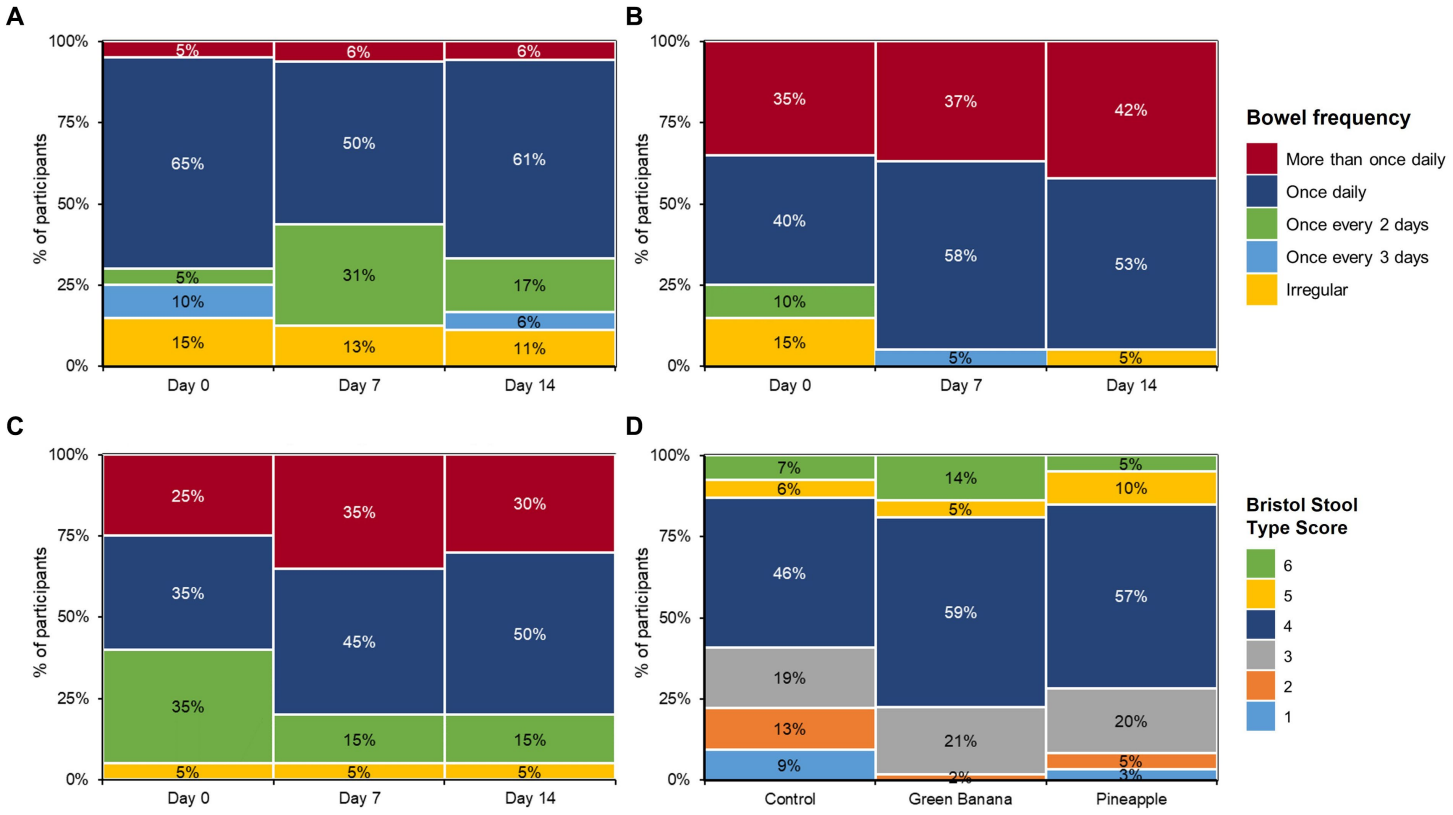
Bowel frequency for **(A)** control, **(B)** green banana, and **(C)** pineapple at day 0, 7, and 14 and **(D)** bowel health across all cohorts.

Control participants had relatively stable more-than-once-daily bowel frequencies between day 0 to 14 (Figure 2A), with slight increases in once-every-2-days frequency and slight decreases in infrequent (irregular, once-every-3-days, and once-daily) bowel movements. In contrast, GBP and PFP improved all frequencies tested, even at early stages (days 0 to 7) of the study. Moreover, GBP and PFP also increased the number of participants with frequent or regular movements. GBP also maintained the improvements in infrequent and irregular movements over the study duration. Notably, those with 7-or-more-per-week average bowel movements increased in more GBP and PFP participants than in controls. In early stages, GBP and PFP increased the number of participants with more-than-once-daily and once-daily frequencies (Figures 2B,C), but control participants remained constant (Figure 2A). There were no reports of constipation or diarrhoea due to GBP or PFP consumption, indicating that daily consumption of 5 g of fibre did not increase gastrointestinal adverse events. After 7 days, normal stool was reported by 65% of control participants versus 79% of GBP participants and 44% of PFP participants. Stools were lumpier in control participants than in GBP or PFP participants. After 7 days of consumption, GBP and PFP also led to softer stools than in control participants. By day 14, stool consistency had normalised (Bristol Type 3 and 4) among GBP and PFP participants but remained unchanged among controls.

### Effects on sleep due to GBP and PFP fibre intake

**Figure 3:**
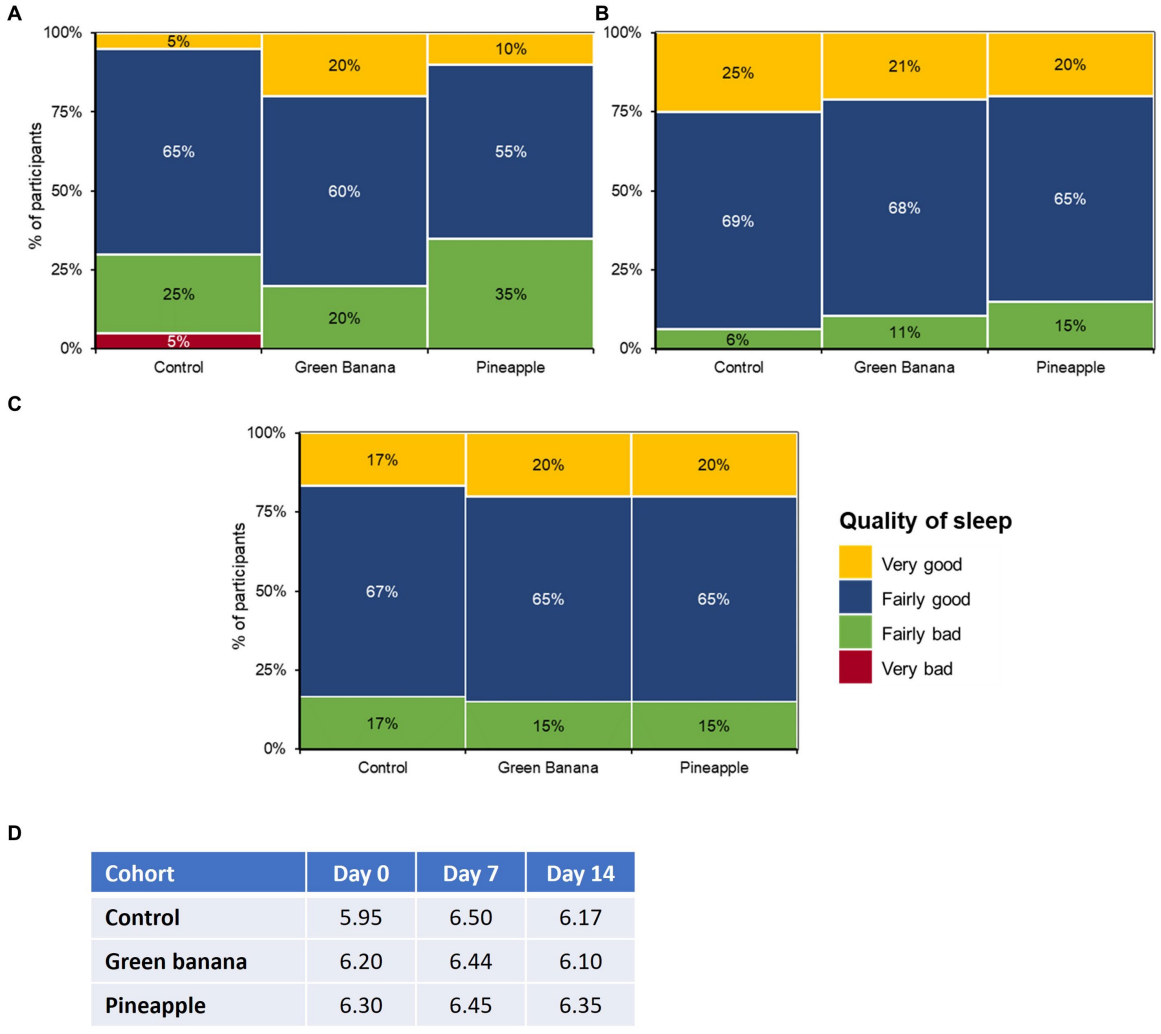
Impact of GBP and PFP on sleep quality at **(A)** day 0, **(B)** day 7, and **(C)** day 14 and **(D)** average sleep hours. Assessments were based on the Pittsburgh Sleep Quality Index (PSQI) survey to measure sleep quality.

Throughout the study, average sleep quality remained unaffected among all cohorts (Figure 3). At day 7, GBP and PFP participants reported “low” experiences of bloatedness and fewer “fairly bad” sleep disruptions due to the additional fibre, with mean hours of sleep remaining constant throughout the study. From days 0 to 7, “fairly bad” sleep disruptions had dropped from 20 to 11% with GBP, and from 35 to 15% with PFP. Throughout the study, the mean hours of sleep remained constant in both GBP and PFP participants but fluctuated among the control cohort.

### Effect of GBP and PFP on the gut microbial composition

**Figure 4:**
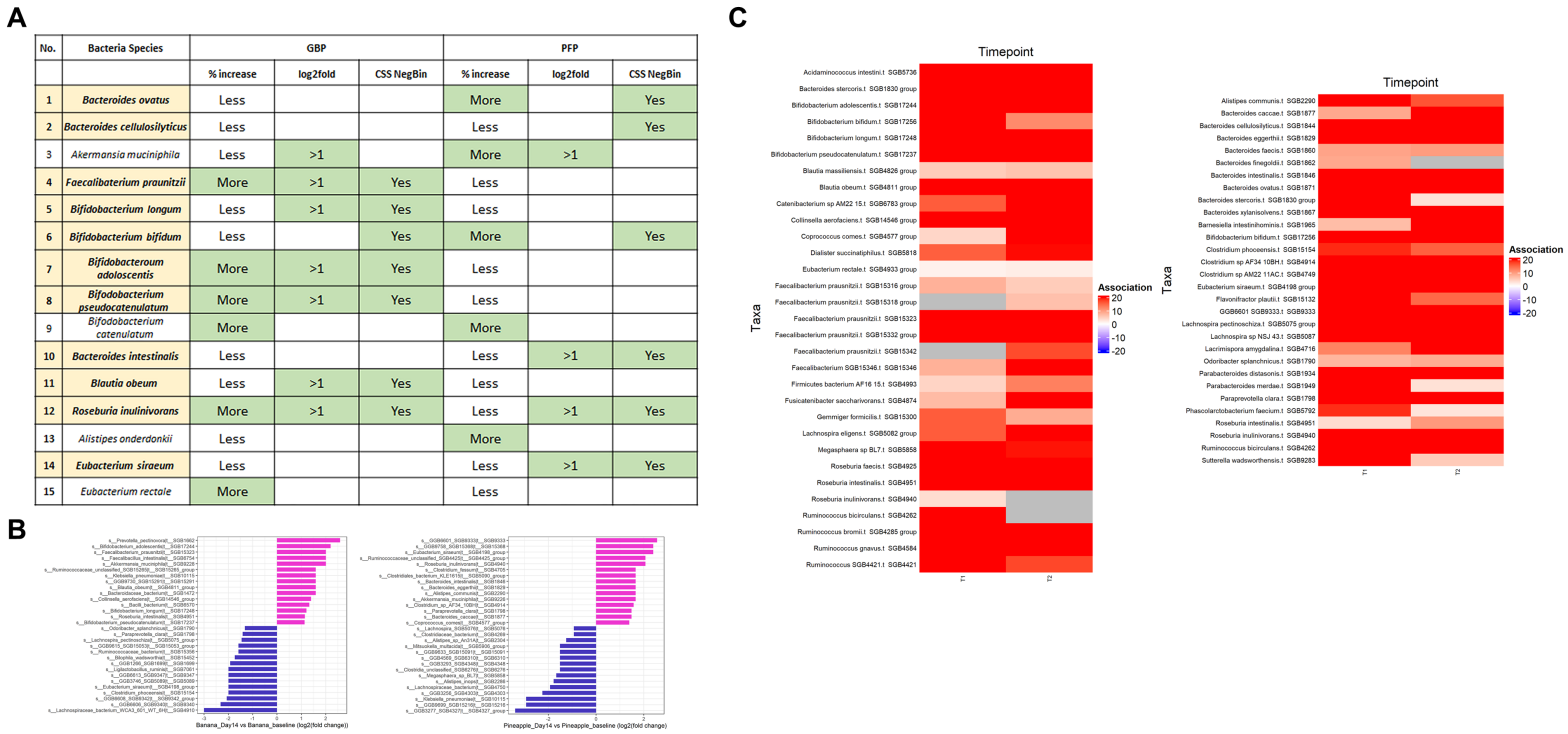
**(A)** Summary of the changes in abundance observed from the 15 beneficial bacteria after supplementation of GBP and PFP. % increase—“Less” = <50% of samples that harboured the species at baseline showed enrichment, “More” = >50% of the samples that harboured the species showed enrichment. **(B)** Log2fold change of top 15 beneficial and disease-associated bacteria between day 14 and day 0 in GBP and PFP groups. Only features with log2FC >1 are shown. **(C)** Significant features under CSS normalised negative binomial model. FDR was set at 0.05.

The prebiotic effect of GBP and PFP were evaluated on 15 health-promoting species (Figure 4). Seven species (i.e., *F. prausnitzii*, *B. longum*, *B. bifidum*, *B. adolescentis*, *B. pseudocatenulatum*, *B*. *obeum* and *R. inulinivorans*) were significantly elevated post-supplementation in GBP while six species (i.e., *B. ovatus*, *B. cellulosilyticus*, *B. bifidum*, *B. intestinalis*, *R. inulinivorans*, and *E. siraeum*) were enriched from PFP supplementation.

### Effect of GBP on gut microbiome

*Bifidobacterium* levels increased significantly from days 7 to 14 among GBP groups, including in Japanese participants, but were similar across timepoints in control groups, including in non-Japanese control participants (Supplementary Figure S2). *F. prausnitzii* was detected in nearly all control and GBP participants and was higher than baseline at day 14 in 40% of control participants and over half of GBP participants. *F. prausnitzii* levels in GBP participants were also higher than baseline at day 7. *R. inulinivorans* was detected in nearly all control participants, 39% of whom had higher levels at day 14 than baseline, and in nearly all GBP participants, less than half of whom had higher levels than baseline. Two participants had very high baselines although these decreased at days 7 and 14. *R. inulinivorans* levels were higher than baseline at day 14 in most Japanese GBP participants. Interestingly, the different cohorts showed variable overall changes in bacterial taxonomic compositions over different timepoints. In control cohorts, the different bacterial abundances were relatively similar at all timepoints, and relatively unchanged in ethnically Japanese or non-Japanese control participants. For instance, on day 7, in both Japanese and non-Japanese control participants, *Bacteroides* increased and then decreased by the same extent. However, *Bacteroides* and *Prevotella* were slightly elevated in non-Japanese control participants at day 7, while other bacteria (e.g., *Ruminococcus*) increased in a time-dependent manner. *Faecalibacterium* uniformly declined at day 7 before increasing at day 14 to almost baseline levels in all control cohorts. In contrast, GBP significantly changed bacterial taxonomic compositions in both a time-dependent and time-independent manner. Notably, GBP consumption significantly increased *Bifidobacterium* across all cohorts in a time-dependent manner from day 0 to 7, with corresponding decreases in *Phocaeicola* and *Alistipes* in all cohorts at all timepoints. A time-dependent increase in *Prevotella* and *Collinsella* was also observed exclusively in Japanese GBP participants.

### Effect of PFP on gut microbiome

Across timepoints, PFP altered bacterial taxonomic compositions, with *B. ovatus* abundance increasing in all ethnicities and *A. muciniphila* increasing specifically in the Japanese (Supplementary Figure S2). Among the notable genera, *Bacteroides* and *Phocaeicola* increased in all cohorts and timepoints (Supplementary Figure S2). Interestingly, *Bifidobacterium* increased at day 7 in both control and non-Japanese PFP participants but dropped slightly at day 14. In contrast, *Bifidobacterium* decreased by day 7 in Japanese PFP participants but increased at day 14, whereas *Prevotella* decreased in all cohorts at all timepoints in a time-dependent manner (Supplementary Figure S2). Although *Bacteroides* abundance declined in all control participants, this was most pronounced in the Japanese. In all ethnicities of PFP participants, *Bacteroides* abundance significantly increased from days 7 to 14. *B. ovatus* was detected in all PFP participants, and at higher levels at day 14 in more PFP participants (including most Japanese) than in control participants. Increased *B. ovatus* levels were seen in half of PFP participants at day 7 and in most PFP participants at either day 7 and/or 14, but a small number of PFP participants had substantially decreased levels over the study duration. *B. cellulosilyticus* was detected in most control participants (a few with levels higher than baseline), in most PFP participants (46% with levels higher than baseline), and at higher levels than baseline in most PFP participants at day 7 and/or 14. *A. muciniphila* was detected in a few control participants, over half of whom had increased levels at day 14 versus baseline. It was also detected in a quarter of PFP participants, most of whom had increased levels at day 7 versus baseline, and in 30% of Japanese PFP participants. Additionally, a few Japanese PFP participants had *A. muciniphila* at day 0, but not by day 14.

### Functional pathways conferred by GBP and PFP

**Figure 5:**
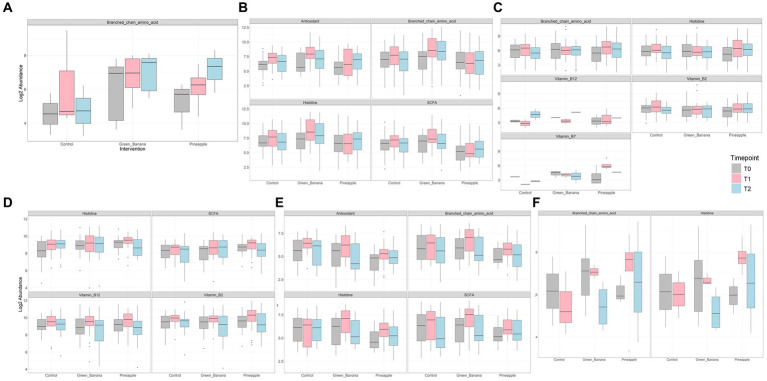
Abundance of key metabolites conferred by microbes **(A)** Akkermansia spp., **(B)** Bifidobacterium spp., **(C)** Bacteroides spp., **(D)** Faecalibacterium prausnitzii, and **(E)** Roseburia inulinivorans after consumption of Green Banana or Pineapple Fiber Powder.

Compared to controls, GBP and PFP consumption increased several beneficial bacteria—*B. longum*, *B. adolescentis*, *B. bifidum*, *F. prausnitzii*, *A. muciniphila*, *B. ovatus*, *B. cellulosilyticus*—across timepoints for all ethnicities, including the Japanese, relative to baseline. GBP and PFP led to increased microbial capacities to produce branched-chain amino acids (BCAA), antioxidants, histidine and SCFA, with PFP also increasing microbial capacities to produce vitamin B7 (Figure 5). GBP and PFP increased *Bifidobacterium* spp. capacity for antioxidant, BCAA, SCFA and histidine production. PFP increased *Bacteroides* spp. capacity to produce BCAA, histidine, vitamin B12, B2 and B7, while GBP increased *Bacteroides* spp. capacity to produce only vitamin B12. Seven days of GBP and PFP consumption increased *F. prausnitzii* capacity to produce histidine, SCFA, vitamin B2 and B12, and *R. inulinivorans* capacity to produce antioxidant, BCAA, histidine and SCFA. Both capacities decreased slightly at day 14. GBP and PFP also increased *E. siraeum* capacity to produce BCAA and histidine at day 4, which diminished slightly at day 14.

### Comparing beneficial species with AMILI database

**Figure 6:**
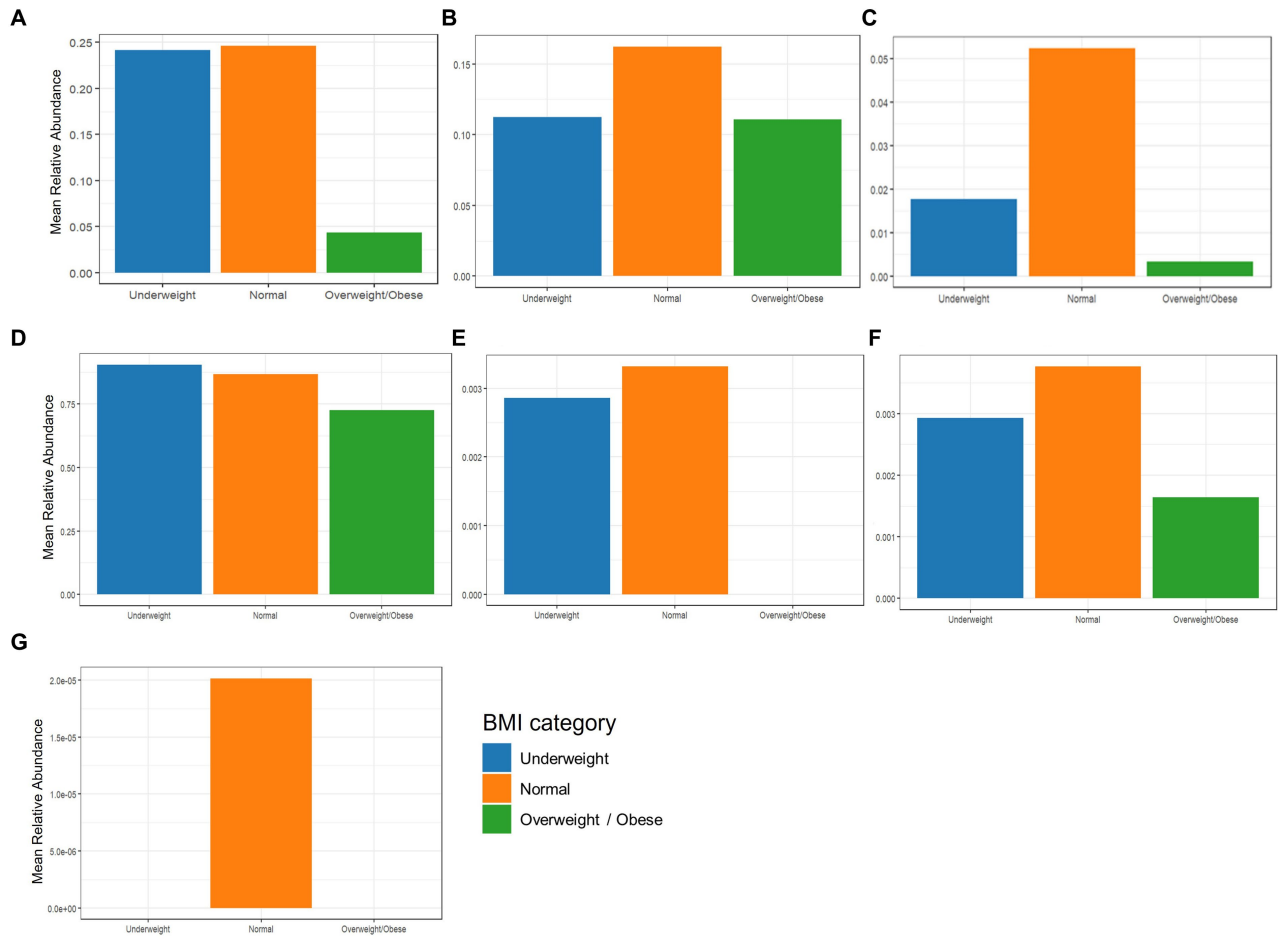
Comparing mean relative abundance of selected beneficial bacteria **(A)** Bifidobacterium longum, **(B)** Bifidobacterium bifidum, **(C)** Bifidobacterium adolescentis, **(D)** Faecalibacterium prausnitzii, **(E)** Roseburia inulinivorans, **(F)** Bacteroides cellulosilyticus, and **(G)** Eubacterium siraeum across BMI categories between the participants’ and subjects in the AMILI database.

**Figure 7:**
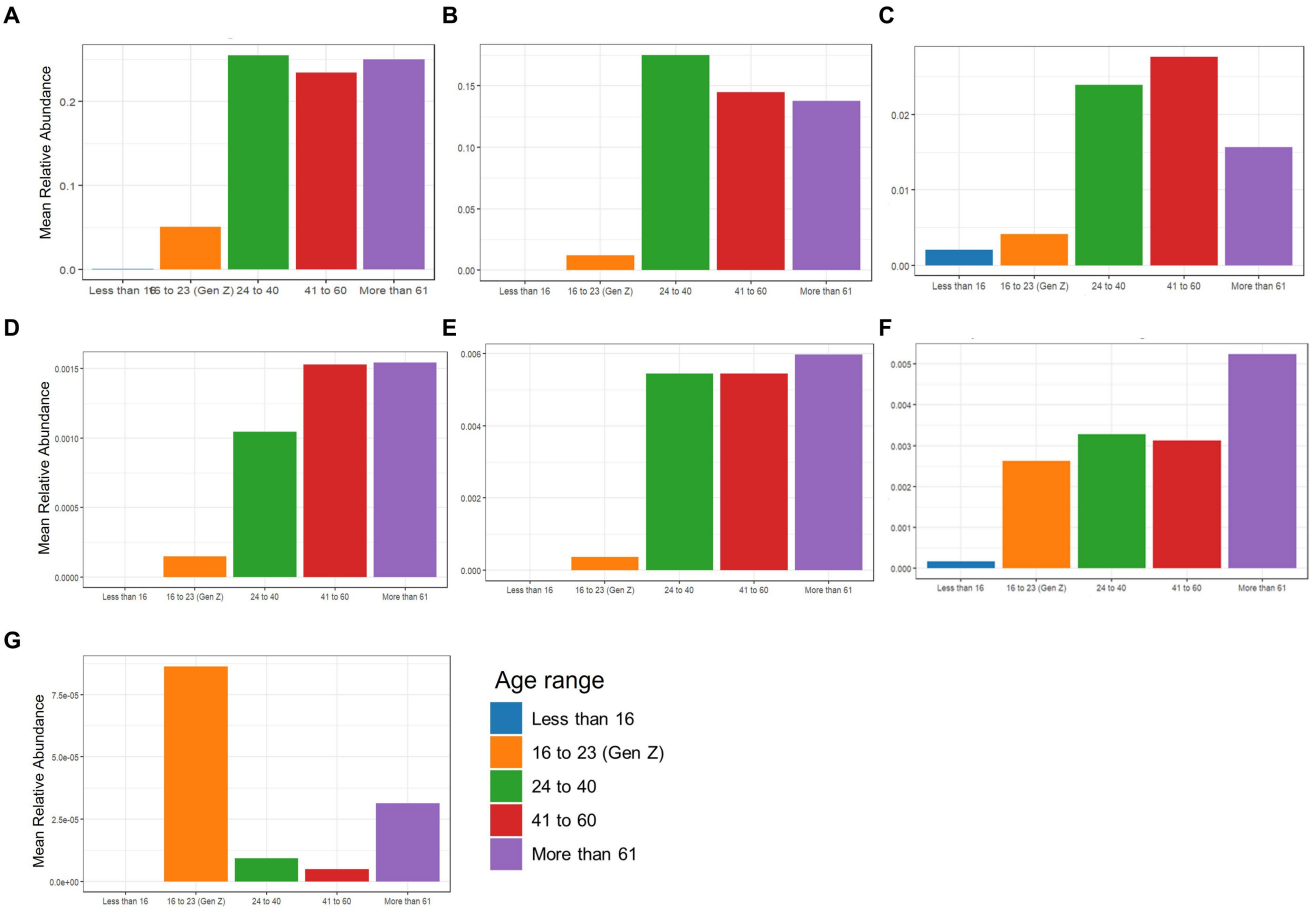
Comparing mean relative abundance of selected beneficial bacteria **(A)** Bifidobacterium longum, **(B)** Bifidobacterium bifidum, **(C)** Bifidobacterium adolescentis, **(D)** Akkermansia mucinphila, **(E)** Bacteroides ovatus, **(F)** Bacteroides cellulosilyticus, and **(G)** Eubacterium siraeum across age range between the participants’ and subjects in the AMILI database.

**Figure 8:**
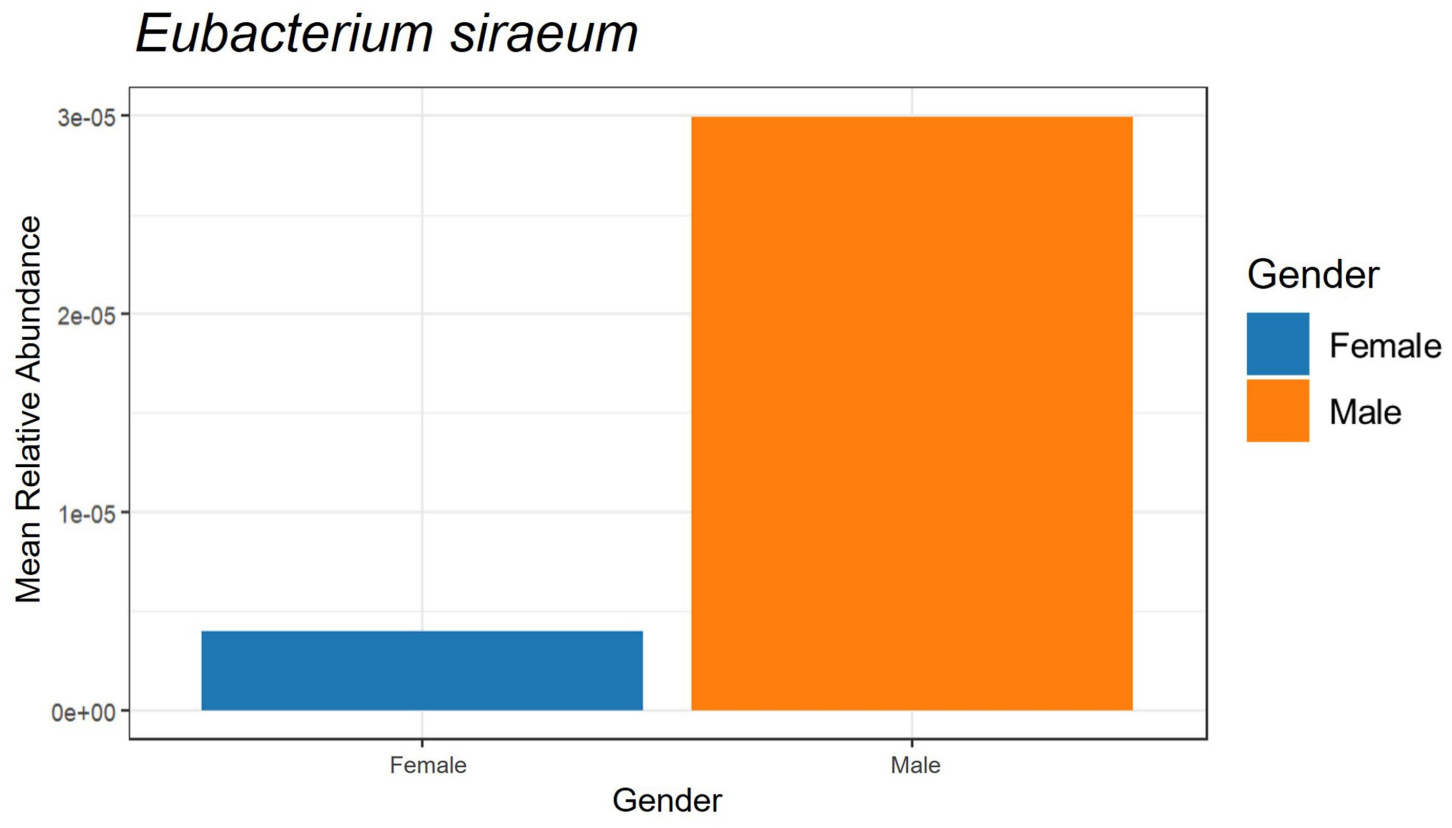
Comparing participants’ genders and beneficial species *Eubacterium siraeum* with subjects in the AMILI database.

The beneficial species detected with GBP and PFP consumption were compared to those from an in-house database (AMILI Pte., Ltd., Singapore). Individuals in the database who were overweight or obese haboured lower abundance of *B. longum*, *B. adolescentis*, *R. inulinivorans* and, *E. siraeum* in comparison to those in the underweight or normal BMI category (Figure 6). *E. siraeum* was also deficient in individuals who were underweight, overweight and/or obese, with females having much lower *E. siraeum* levels than males. Since GBP increased the abundance of these taxa, overweight and/or obese females are likely to benefit the most from GBP consumption. Individuals with less *B. longum*, *B. adolescentis*, *A. muciniphila*, *B. ovatus*, and *B. cellulosilyticus*, were aged 16 and younger, or 16 to 23 years (Figure 7). Those aged 24 to 60 years lacked *E. siraeum* the most, especially females (Figure 8). As PFP consumption increased these microbes in both genders aged 16 to 23 years, these individuals are likely to benefit the most from PFP consumption.

## Discussion

We evaluated the effects of fibre-containing powders from two common tropical fruits—green bananas and pineapples. By recovering and reusing fruit processing waste, we produced fibre-rich supplements with health benefits, in agreement with previously published data (16, 20, 23). Both powders improved gut health by increasing the levels of beneficial microbes, and improved metabolism by promoting the production of histidine, BCAA, SCFA, and biotin. Moreover, PFP benefitted both genders aged 16 to 23 years, while GBP benefitted overweight/obese individuals, including females.

GBP and PFP are unique dietary fibre supplements due to the advanced technologies used to process fruit wastes and generate these powders. The environmentally friendly, minimal processing methods retains the fruits’ maximum nutritional benefits. Simultaneously, it ensures compatibility between the powders’ physical and chemical properties, and their intended food and beverage applications. Unlike most processed fibre products, no artificial additives are used to preserve the fruits’ health benefits. Our physical analysis of GBP and PFP showed both to have the sensory properties of a free-flowing powder and to solubilise easily with liquids to facilitate ingestion. Additionally, their colour and odour were characteristic of their source fruits, and thus familiar and palatable to consumers (14). Our post-processing nutrient analyses noted 77.4 g of dietary fibre per 100 g of GBP, and 69.1 g of dietary fibre per 100 g of PFP. Among the total starches present in GBP, 38.1% was comprised of RS.

Consumption of the additional GBP and PFP dietary fibre and RS promoted temporal increases in various beneficial gut microbial species and increased their diversity, in many instances across timepoints and ethnicities. In agreement with studies reporting green banana pulp and by-products to benefit diarrhoea and constipation (13, 48), our study showed that GBP improved bowel movement frequencies and stool consistencies in most participants after just 1 week. Likewise, PFP significantly improved microbiome compositions and increased the levels of various microbes across timepoints and ethnicities. Additionally, the dietary fibre in PFP was the likely reason for 44% of PFP-consuming participants reporting normal stool consistencies. Other studies have similarly found pineapple powders to give prebiotic effects on microbes such as *Lactobacillus* (16), or increased growth of *Lactobacilli* and *Bifidobacteria* (23). Therefore, GBP and PFP dietary fibre and RS promoted improved bowel regularity and overall bowel health. Our findings align with others showing that gastrointestinal symptoms were reduced and improved by consuming a novel RS blend (RSB) formulated from green banana flour and apple pectin, which increased the levels of the beneficial microbes, *Alistipes* and *Faecalibacterium* (20). Moreover, gut bacteria can ferment or metabolise RS, carbohydrates (glycans) and diverse fibres (e.g., cellulose, pectins, fructans) that are resistant to human enzymes (1). GBP and PFP thus offer a high diversity of fibres and probiotics to these bacteria, and positively modulate the gut microbiome.

As our data shows, GBP and PFP increased the abundance and diversity of beneficial bacteria, and promoted these microbes’ overall functional ability to produce BCAAs, antioxidants, histidine, SCFA, and B vitamins. Butyrate, which is nutrition-dependent (30) and affected by prebiotics, is necessary for human gut health and physiology as it provides energy for cells in the colon and intestines (1), maintains gut wall integrity, and is needed for mucosal immunity and overall intestinal homeostasis. BCAAs (e.g., histidine) obtained through diet or *de novo* gut bacteria synthesis (49), modulate gut health and regulate energy homeostasis, metabolism and immunity. Low BCAA levels may signal the risk or presence of several disorders and diseases, including obesity and insulin resistance, Parkinson’s disease, and metabolic-associated fatty liver disease or non-alcoholic fatty liver disease (NAFLD). Host-derived histidine is a critical energy source for microbiomes and has antioxidant roles (49, 50). Low circulating histidine levels are seen in chronic kidney disease and heart failure (49). Consuming 4.0–4.5 g histidine/day leads to glucose homeostasis, pro-inflammatory cytokines, lower BMI and adiposity, and improved cognitive function (50). In obese women with metabolic syndrome, histidine supplementation improves oxidative stress, inflammation, and insulin resistance. B vitamins such as biotin (vitamin B7) prevent intestinal inflammation and contribute to the overall health of the gut ecosystem. Other studies on pineapple-derived polyphenols from pineapple snack bars reported modulation of multiple bacterial species and their production of metabolites (propionate, acetate). In turn, this regulated antioxidant and anti-inflammatory effects that reduced anxiety and depression (23).

The gut microbiome may use the gut-brain axis to affect endocrine, neural, and immune pathways, and modulate sleep (51). Consumption of more fibre increases the SCFAs produced by gut microbes. In turn, this stimulates the production of sleep cytokines and serotonin while blocking inflammatory pathways, thereby ameliorating sleep disorders. RSB was associated with *Bacteroides* (20), which produces gamma aminobutyric acid (GABA) and may benefit sleep (52). Across cohorts throughout our study, the additional GBP or PFP fibre caused “low” experiences of bloatedness and reduced “fairly bad” sleep disruptions, without impacting sleep durations. Large cohort studies with longer durations and sleep monitoring devices are needed to identify specific associations between changes in sleep-related factors and bacterial strains or species.

GBP consumption may also be particularly beneficial for females lacking *E. siraeum*, who, according to the AMILI database, is associated with individuals whose BMI falls into the overweight or obese category. *E. siraeum* is significantly decreased in several conditions and diseases, including metabolically healthy obesity. Higher levels of this bacteria and its metabolites (53), have been linked to reduced gastric mucosal damage, lower faecal calprotectin levels, and improved chronic superficial gastritis (54).

*B. longum*, *B. adolescentis*, *B. bifidum*, *A. muciniphila*, *B. ovatus*, *B. cellulosilyticus*, and *F. prausnitzii*, are beneficial bacteria promoted by GBP and PFP. However, these bacteria were found to be deficient in younger individuals (16 to 23 years). PFP may thus increase these microbes’ abundance and benefit these individuals the most. For example, *A. muciniphila* (55) maintains gut barriers and regulates inflammation. *A. muciniphila* levels correlate positively in overweight and obese individuals undergoing caloric restriction, with improved outcomes and healthy metabolic status. Conversely, its absence or reduction is linked to obesity, diabetes, and ulcerative colitis. *F. prausnitzii* (56) is seen in intestinal disorders, obesity, metabolic syndrome or NAFLD, and is present at low levels in elderly and Westernized populations. IBD sufferers who consume *B. ovatus* (57) have increased SCFAs, which modulates gut homeostasis and development, and regulates lipid and glucose metabolism (58). Intestinal *B. ovatus* can also increase levels of neuro-active SCFAs. High *B. cellulosilyticus* levels are significantly correlated with healthy, plant-based diets, but is depleted in MHO and atherosclerotic cardiovascular disease. Its intake lowers plasma lipids while improving cardiac function and atherosclerotic plaque formation (59). *Bifidobacteria* produce metabolites including GABA and SCFAs that protect from infection and microbiome imbalances and participate in immune responses. *B. bifidum* modulates intestinal permeability and lipid metabolism and protects from inflammation. It also inhibits liver fat deposits and inflammation. In several gastrointestinal diseases (e.g., Crohn’s disease), *B. bifidum* reduces abdominal pain, dyspepsia, and disease severity (60). *B. longum* also generates beneficial SCFAs and bioactive substances that restore mucus layers in colon barrier defects caused by Westernized diet-associated microbiome changes (61). Its levels are inversely related to insulin and insulin resistance in obesity and decreased by high glucose and insulin levels. It reduces pro-inflammatory cytokines and inflammation, improves chronic inflammation, and regenerates epithelial tissues, thus improving gastrointestinal conditions (62). Intestinal *B. adolescentis* is significantly decreased in type 2 diabetics, and its intake can lower blood glucose levels (63). Although not well studied, *B. catenulatum* is increased in infant allergies and in adults, but low in inflammatory bowel syndrome (64). It can have immunomodulatory and anti-inflammatory properties, improve colitis, treat systemic inflammation and some acute liver injuries, and address dysbiosis of the gut microbiome, which refers to the imbalance or disequilibrium of gut microbial communities.

Future studies should focus on the specific effects of dietary fibre supplementation on gut health across different population groups. Targeted research on GBP in overweight and/or obese individuals could provide insights into metabolic and microbiome responses, while exploring the impact of PFP in young adults aged 16 to 23 years could elucidate its potential benefits in this demographic. Additionally, investigations into the long-term effects of these ingredients and their interactions with diverse dietary habits will be crucial in tailoring effective, personalised gut health interventions. Nevertheless, GBP and PFP lead to the production of beneficial microbes and supported their growth or function, indicating that dietary fibre supplement with GBP and PFP may substantially benefit human health.

## Conclusion

**Figure 9:**
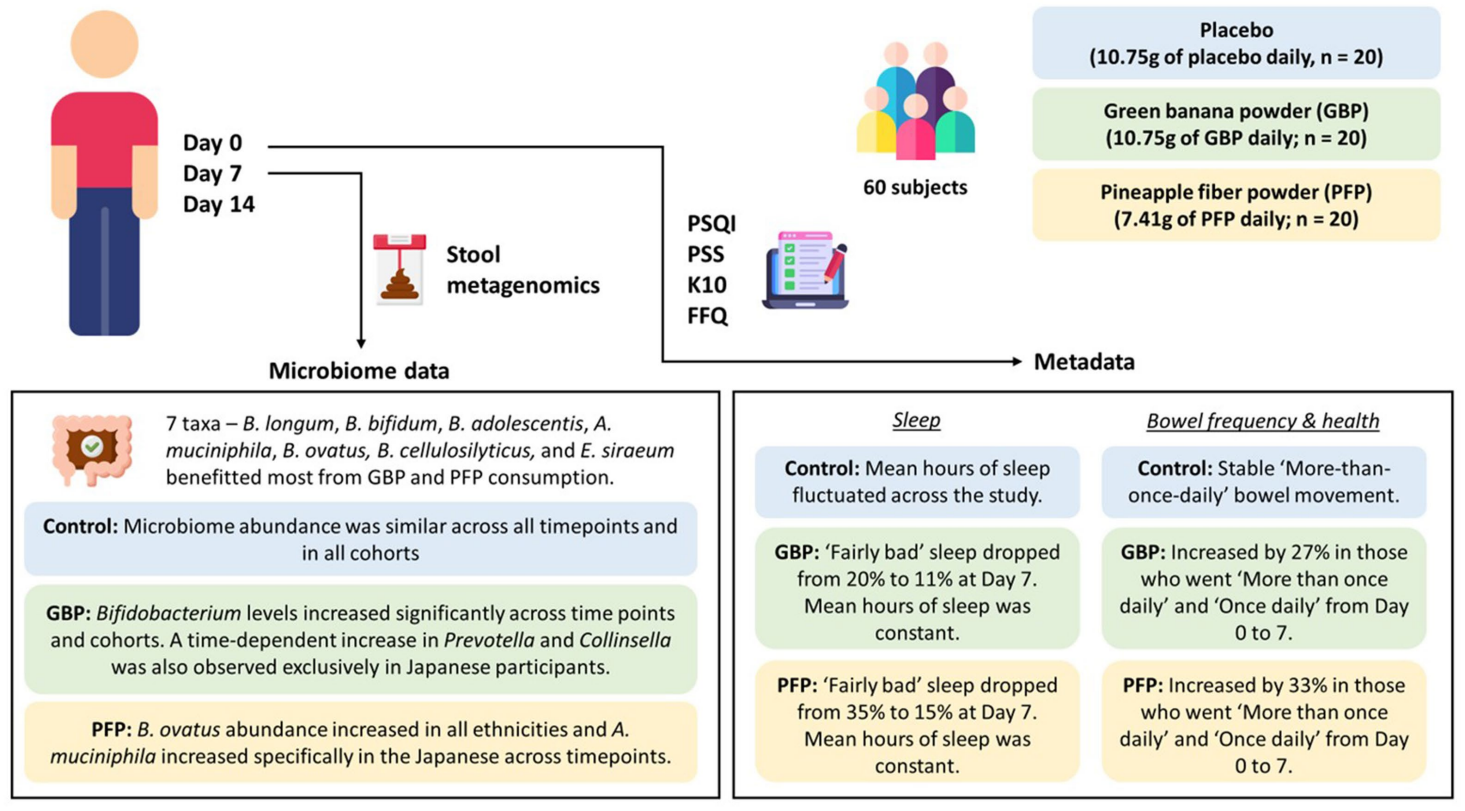
Summary of changes in microbial abundance and the impacts on sleep, bowel frequency and stool health with GBP or PFP consumption. (PSQI, Pittsburgh Sleep Quality Index; PSS, Perceived Stress Scale; K10, Kessler Psychological Distress Scale; FFQ, Frequency Questionnaire; GBP, green banana powder; PFP, pineapple fibre powder).

Although our study was brief and limited by a small sample size, lack of age and ethnic diversity, short durations, and inter-individual variations, we found that dietary supplementation with GBP and PFP, which are enriched in insoluble dietary fibres, led to increases in beneficial bacteria numbers and metabolites, and improved host gut health (Figure 9). While the varied microbial responses to these substrates might be attributed to the fruits’ variable fibre compositions, our database comparisons indicate that GBP and PFP confer substantial benefits to the human microbiome. In particular, GBP may benefit overweight and/or obese individuals, including females, while PFP may benefit young adults aged 16 to 23 years regardless of gender. In conclusion, dietary supplementation with GBP and PFP should be considered a key nutritional strategy to address inadequate fibre consumption and to improve human health quickly, conveniently, and efficiently.

## Data Availability

The datasets presented in this study can be found in online repositories. The names of the repository/repositories and accession number(s) can be found at: https://www.ncbi.nlm.nih.gov/genbank/, 1115893.

## Supplementary material

SUPPLEMENTARY FIGURE S1 Alpha diversity indices for overall (top) and Japanese only (bottom) participants across time points for control, GBP, and PFP.

SUPPLEMENTARY FIGURE S2 Effect of PFP (top) and GBP (bottom) on gut microbiome with changes in alpha diversity between cohorts across time points. Top 20 species in Japanese and non-Japanese control cohorts (**A,D** in top and bottom), in Japanese participants only (**B,E** in top and bottom), and in non-Japanese participants only (**C,F** in top and bottom).

SUPPLEMENTARY TABLE S1 Nutrient profile of green banana powder (GBP) and pineapple fiber powder (PFP).

SUPPLEMENTARY TABLE S2 Demographic breakdown of subjects from the AMILI database (*n* = 669).
