# Supplementary figures and images for: Effect of green banana and pineapple fibre powder consumption on host gut microbiome
Source: Front Nutr. 2024 Aug 23;11:1437645. doi: 10.3389/fnut.2024.1437645 (PMC11378528; doi:10.3389/fnut.2024.1437645)

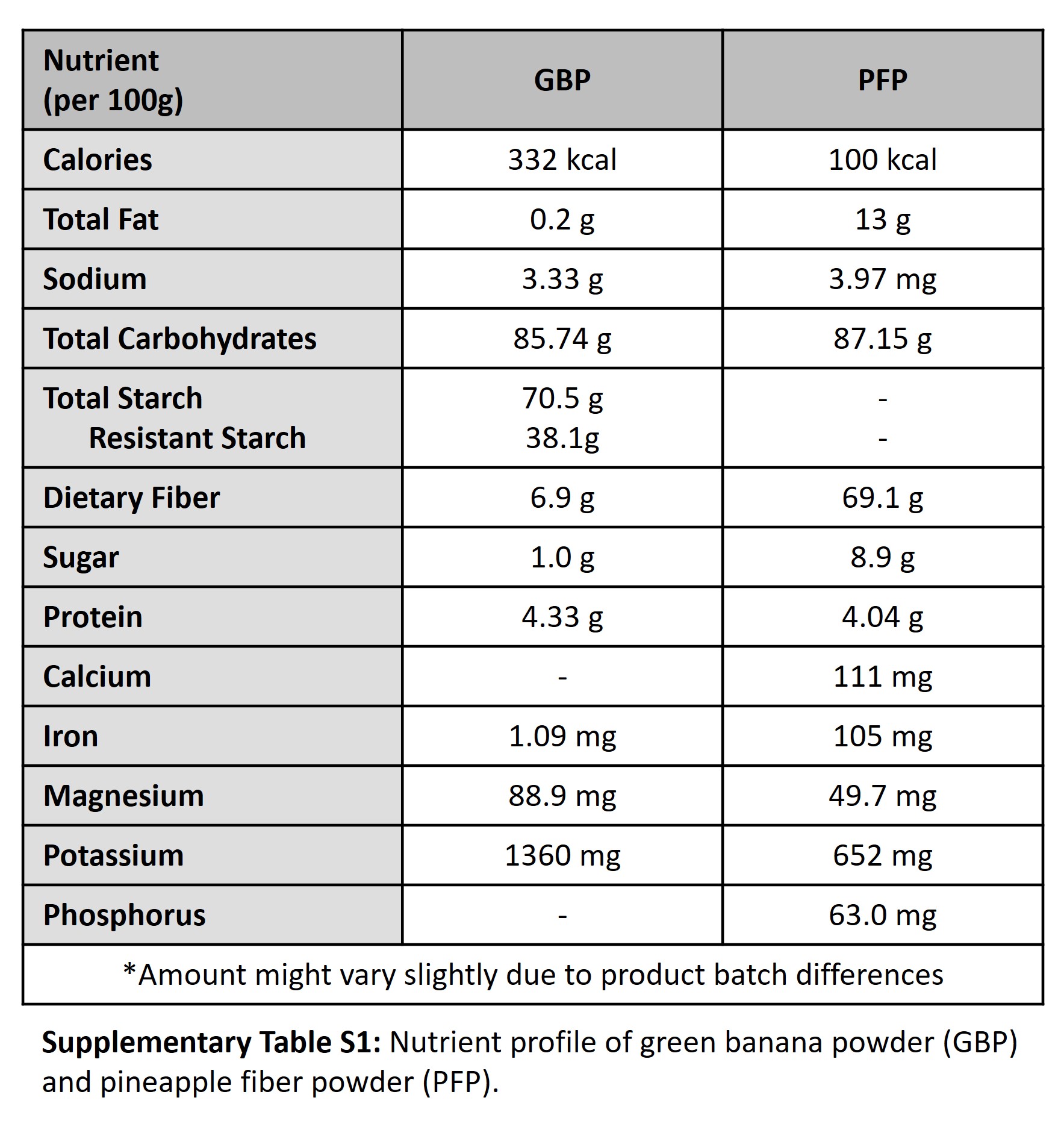

Supplement: SUPPLEMENTARY FIGURE S1 — Alpha diversity indices for overall (top) and Japanese only (bottom) participants across time points for control, GBP, and PFP. [file Image_1.jpg]

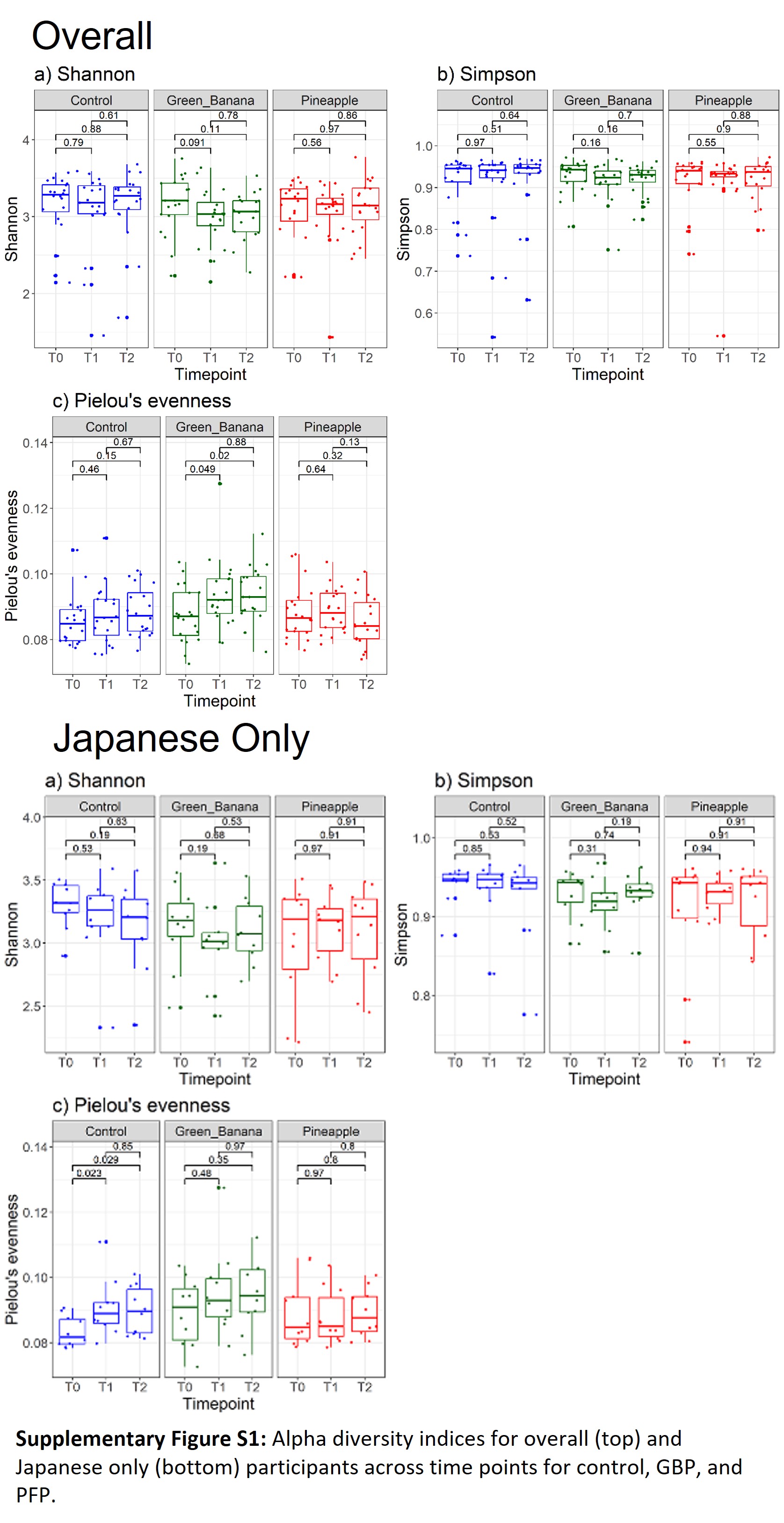

Supplement: SUPPLEMENTARY FIGURE S2 — Effect of PFP (top) and GBP (bottom) on gut microbiome with changes in alpha diversity between cohorts across time points. Top 20 species in Japanese and non-Japanese control cohorts (A,D in top and bottom), in Japanese participants only (B,E in top and bottom), and in non-Japanese participants only (C,F in top and bottom). [file Image_2.jpg]

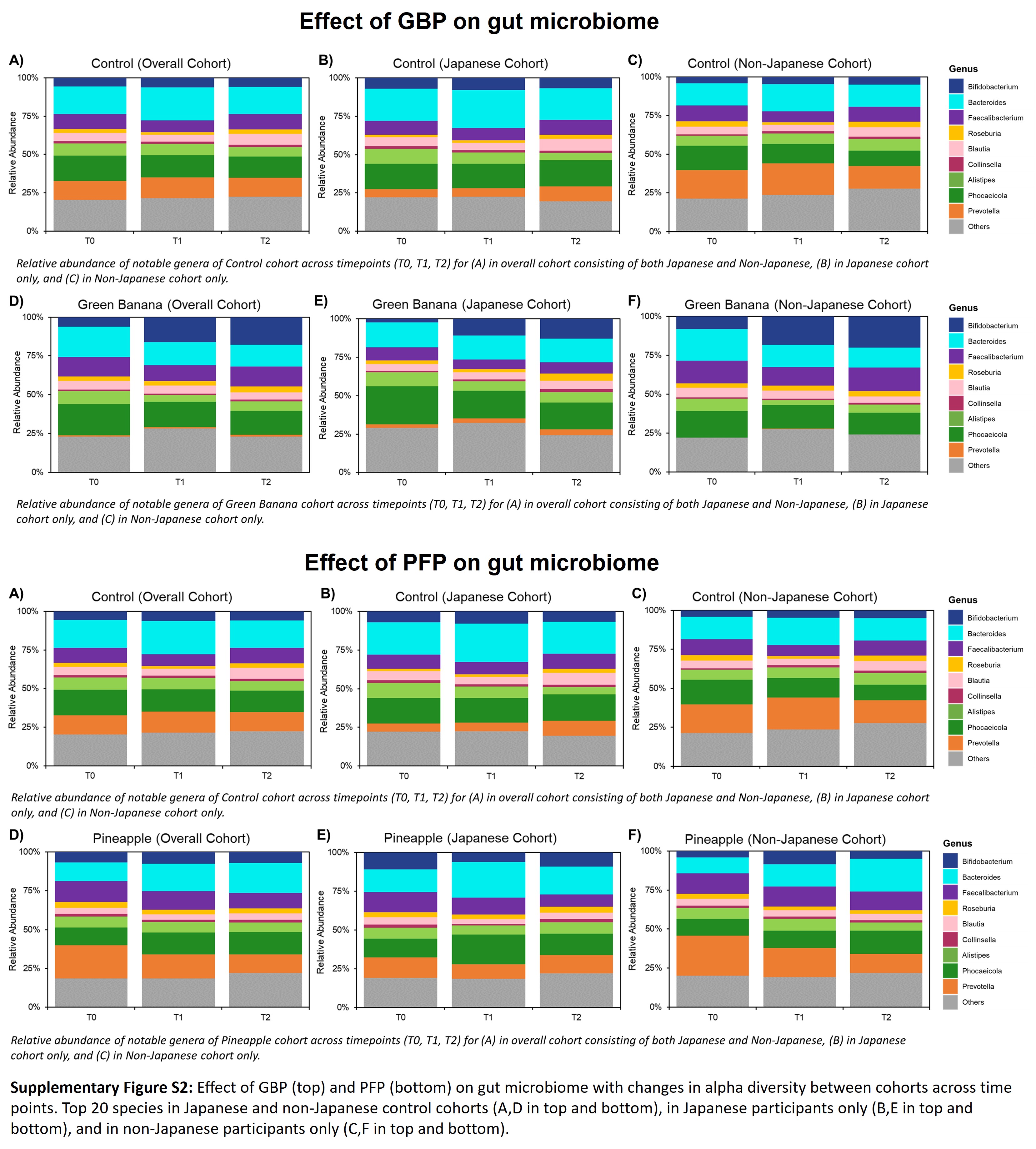

Supplement: SUPPLEMENTARY TABLE S1 — Nutrient profile of green banana powder (GBP) and pineapple fiber powder (PFP). [file Image_3.jpg]

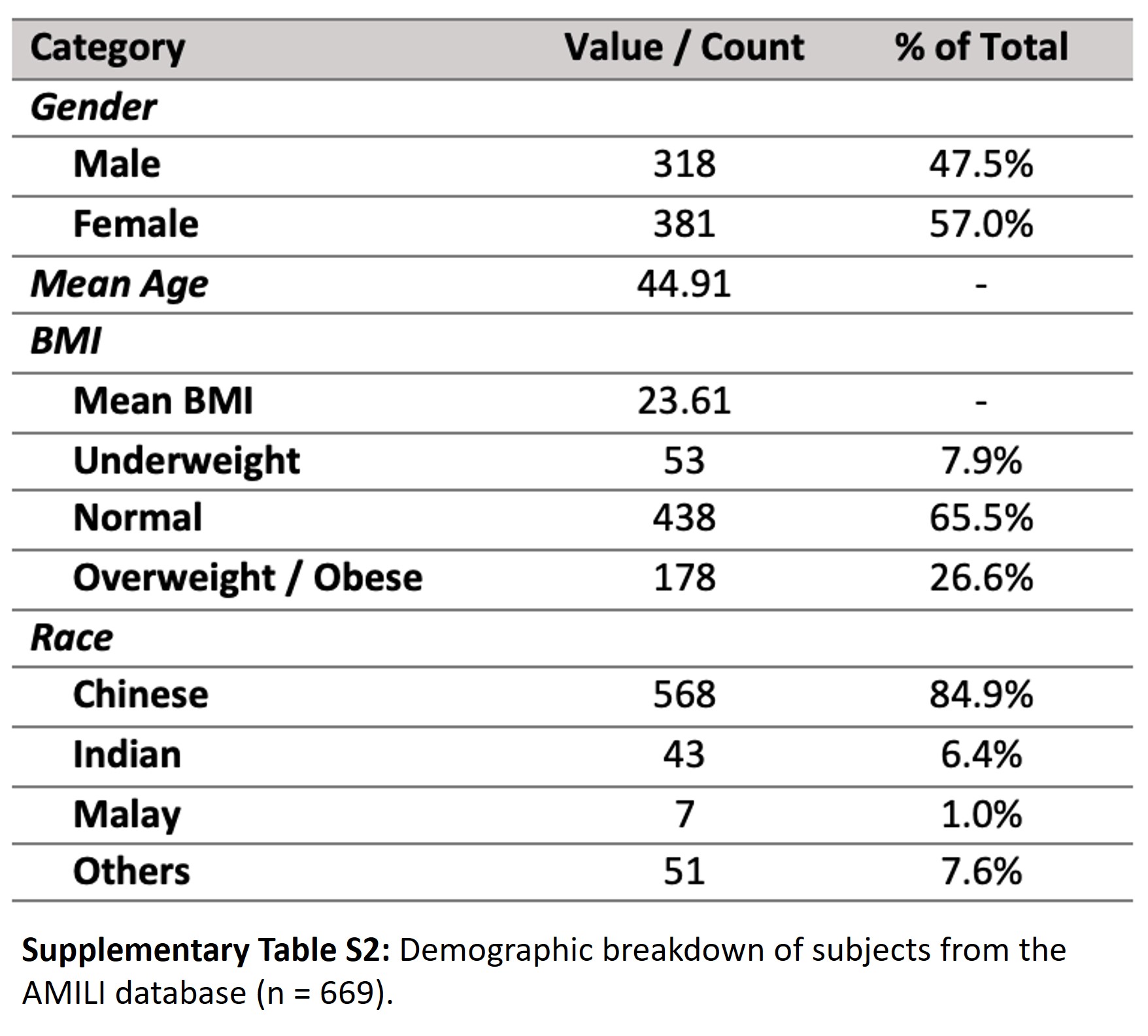

Supplement: SUPPLEMENTARY TABLE S2 — Demographic breakdown of subjects from the AMILI database (n = 669). [file Image_4.jpg]
